# Qualineo Strategy Indicators Associated with Neonatal Death: A Cross-Sectional Study

**DOI:** 10.3390/ijerph21081096

**Published:** 2024-08-19

**Authors:** Camila Evangelista de Sousa Campelo, Cynthia Roberta Dias Torres Silva, Rejane Corrêa Marques, Ana Maria Ribeiro dos Santos, Nathaly Marques Santos Machado, Márcia Teles de Oliveira Gouveia

**Affiliations:** 1 Nursing Graduate School, Universidade Federal do Piauí, Teresina 64049-550, Brazil; camila.oliveira@ufpi.edu.br (C.E.d.S.C.); rejanecmarques@macae.ufrj.br (R.C.M.); anasantos@ufpi.edu.br (A.M.R.d.S.); nathaly.santos@ufpi.edu.br (N.M.S.M.); 2 Instituto Federal do Piauí, Teresina 64000-040, Brazil; cynthia.silva@ifpi.edu.br

**Keywords:** infant mortality, newborn, perinatal care, maternal and child health services, neonatal nursing

## Abstract

Context: The Qualineo Strategy is an effective measure for reducing neonatal mortality in regions with the highest death rates. In addition, it is a relevant Brazilian tool for strengthening teamwork and neonatal assistance. This study aims to analyze the predictors of neonatal death in the indicators of care provided by the Qualineo Strategy at a reference maternity hospital in Piauí, in the years 2021 to 2022. Methods: This is a retrospective study of 1856 newborn records. Pearson’s chi-squared test was used to assess the association between the variables; a predictive regression model was used to identify the variables that predict neonatal mortality. Results: There was a significant association between all neonatal variables and the outcome of death (*p* < 0.05). The predictor variables for death in term newborns were the use of drugs by the mother and admission to the Neonatal Intensive Care Unit. For premature newborns, the predictor variables were, as follows: the use of cannula ventilation, an Apgar score in the 1st minute <7; and admission to the Neonatal Intensive Care Unit. Conclusions: The results will make it possible to visualize better strategies for the reality analyzed and reinforce the importance of prenatal care.

## 1. Introduction

Continuous monitoring of the infant mortality rate, including the neonatal component, is of great importance to health in national and international pacts. In this context, the United Nations (UN) launched the Sustainable Development Goals (SDGs) for the period 2016 to 2030, including the need to reduce neonatal and infant mortality in SDG 3, regarding health and well-being, as this is a reliable indicator of weaknesses in relation to socioeconomic conditions, public policies, and the performance of health services [1].

The advancement of public health programs and policies around the world has resulted in changes to newborn care with the aim of improving care and reducing neonatal morbidity and mortality rates (0 to 27 days of life). Neonatal deaths, and post-neonatal deaths (28 days to under one year), are the basis for calculating infant mortality, which in turn is an important indicator for assessing quality of life and the population’s access to health services [2,3].

The global challenge set by SDG 3 is to reduce neonatal and infant mortality to 12 deaths and 25 deaths per 1000 live births, respectively. In the Brazilian scenario, the target has been adjusted to five neonatal deaths per thousand live births by 2030. However, Brazil faces obstacles in relation to preventable causes, especially those that can be reduced through care for women during pregnancy, childbirth, or for the newborn. This scenario is worse in the north and northeast regions of the country, where neonatal mortality rates are more pronounced, which indicates the need for qualified health care and assistance [1,4].

In relation to the neonatal mortality rate (NMR), Brazil showed a reduction from 25.33 per 1000 live births in 1990 to 8.5/1000 live births in 2019. In the same timeframe, developed countries such as the United States and Canada, had an NMR of 3.6/1000 live births and 3.4/1000 live births, respectively (World Health Organization, 2019). On the national scene, Piauí has one of the highest neonatal mortality rates in Brazil, with rates above 10.0/1000 live births in 2017, behind only Amapá (13.9/1000 live births), Sergipe (11.9/1000 live births), Bahia (11.8/1000 live births), Pará (11.7/1000 live births), Maranhão (11.7/1000 live births), and Amazonas (10.7/1000 live births). In the same year, states in the south and southeast, such as Santa Catarina (7.4/1000 live births and Espírito Santo (7.6/1000 live births), had lower rates, reinforcing discrepancies between the regions in Brazil and the need for targeted interventions [5].

Although there has been a reduction in neonatal death rates, there are still discrepancies between Brazilian regions, with the north and northeast having the highest rates. Given this reality, the Ministry of Health created the Qualineo Strategy in 2017 with the aim of reducing neonatal mortality and improving newborn care in nine maternity hospitals in the states with the highest neonatal death rates in the country: Amapá, Amazonas, Bahia, Maranhão, Mato Grosso, Pará, Piauí, Roraima, and Sergipe. The initiative, since its implementation, has brought positive results in the reduction in neonatal deaths, as confirmed by the 7.49% reduction in mortality between 2019 and 2020 in a reference maternity hospital in the state of Piauí, in the Northeast region [6].

The Qualineo Strategy contains the main topics to ensure better health conditions for newborns, and its main challenge is the integration of actions that are usually offered and monitored in isolation, such as the qualification of neonatal beds, the Baby-Friendly Hospital Initiative (BFHI), Kangaroo Mother Care for Low-Birth-Weight Babies, Human Milk Banks (HMB), infection prevention, neonatal resuscitation, and safe hospital discharge [7].

One of the tools in this strategy is the neonatal care monitoring form, which contains various topics related to the newborn’s health that can be associated with the death outcome, such as maternal and birth data that include, as follows: information on the presence of maternal comorbidities; length of rupture of membranes; Apgar score at the 1st and 5th minute; the need for newborn resuscitation; hospitalization in neonatal units; infection; nutrition; and congenital anomalies. The analysis of this data makes it possible to identify the main risk factors that may have contributed to the death of the newborn, and thus subsidize actions aimed at changes in care [7].

The Qualineo Strategy is relevant because it is a tool that involves multi-professional care in the face of maternal and neonatal demands. In this sense, teamwork is strengthened in order to improve neonatal care and reduce the health risks that lead to death. The role of nurses is of great importance to the effectiveness of the Qualineo Strategy because they provide care in different contexts that impact the health of newborns such as prenatal care and childbirth, and care based on scientific evidence for premature newborns and those admitted to neonatal units. In addition, health professionals are the ones who can most effectively identify risk factors associated with newborn morbidity and mortality [8,9].

The aim of this study was to analyze the predictors of neonatal death in the care indicators carried out by the Qualineo Strategy at a reference maternity hospital in Piauí, in the years 2021 to 2022.

## 2. Materials and Methods

### 2.1. Study Design and Participants

This is an analytical, cross-sectional, and retrospective study analyzing neonatal monitoring records from January 2021 to December 2022. The study design nomenclature was classified as retrospective because the records were kept before the date of data collection. In this type of study, it is essential that there is credibility in the data recorded in relation to the exposure and intensity of the factors studied, which is a limiting factor for the development of the study [10]. Data collection took place from May to July 2023. The study was carried outat a public maternity hospital in the capital of the state of Piauí, located in the north-central region of Piauí in northeastern Brazil. The study took place at the institution’s Maternal and Neonatal Death Surveillance Center, the sector responsible for storing, typing, and analyzing the neonatal monitoring forms of the Qualineo Strategy, as well as for identifying the determinants of maternal, infant, and neonatal deaths. The forms of the Qualineo Strategy were collected from the following neonatal units: Neonatal Intensive Care Unit, Conventional Neonatal Intermediate Care Unit, and the Kangaroo Intermediate Care Unit.

### 2.2. Sample and Data Collection

The study population consisted of 1856 newborns admitted to neonatal units at the high-risk maternity hospital in the municipality of Teresina, Piauí, between January 2021 and December 2022, whose neonatal monitoring forms of the Qualineo Strategy had been completed. The sample was non-probability (sequential) and consisted of all the monitoring forms from that timeframe. Therefore, the study included data from the neonatal care monitoring forms that were entered during the period defined for the study. No form was excluded, as this would have significantly reduced the sample and would have hindered the analysis. However, all the forms analyzed had at least 88% of the data filled in. It was not possible to identify the missing data from the neonatal monitoring forms due to the impossibility of accessing the manuscripts of the Qualineo monitoring forms in their entirety.

### 2.3. Study Variables

The source of information was secondary data collected from the neonatal care monitoring forms. The form is made up of the following items: identification of the record, maternal data, birth data, hospitalization in the branches of the neonatal unit, kangaroo method, respiratory system, brain and abdomen, infection, vascular access after admission to the neonatal unit, nutrition, retinopathy of prematurity, congenital anomaly, and outcome. The information on the form was entered into Microsoft Excel spreadsheets and then entered into the Brazilian System for Monitoring Obstetric and Neonatal Care, run by the Oswaldo Cruz Foundation. For data collection, the spreadsheets were organized and transformed into a database after coding the variables.

The dependent variable used was neonatal death. The independent variables were grouped into two domains: the mother’s social and clinical profile, which included items related to the mother’s age, skin color, schooling, drug intake, clinical complications related to childbirth and type of delivery; and the newborn’s clinical profile, which included the variables gender, birth weight, gestational age, use of resuscitation, time to umbilical cord clamping, hospitalization in neonatal units, and infection.

In addition, the variables were presented in a predictive model of neonatal mortality at the local health institution in the study, which allowed for the identification of a pattern of occurrence and prediction of potential pregnant women for health and well-being programs, with an impact on the safety of the patients cared for and the care costs employed in one of the most important reference services in the state of Piauí.

### 2.4. Data Analysis

Data analysis was carried out using Stata^®^ software (Statacorp, College Station, TX, USA), version 14. Pearson’s chi-squared test was used to assess the association between the study variables, and the prevalence ratio (PR) was calculated with a 95% confidence interval, estimated by Poisson regression to measure the strength of association between the dependent variable and independent variables. The significance level adopted for the tests was *p* < 0.05. Multivariate analysis was used to identify the variables associated with neonatal death, stratified according to whether the newborn was full-term or premature. For each model, all the variables with a *p*-value < 0.05 in the bivariate analysis were selected for the multivariate Poisson regression and inserted simultaneously. Then, all those with a *p*-value > 0.05 were removed (backward). Only the variables with a *p*-value < 0.05 were kept in the final model.

### 2.5. Ethical and Legal Aspects of Research

This research followed the ethical guidelines of Resolution 466/2012 of the Brazilian National Health Council and was approved by the Research Ethics Committee of the Federal University of Piauí, Brazil, under Opinion number: 5.706.053. We ensured absolute confidentiality of the data collected and the collection institution by not passing on all or part of the records to people not involved in the research team.

## 3. Results

Table 1 shows the PR of maternal social and clinical profile with the outcome of death in the time period analyzed. It was observed that mothers with 8 years or more of schooling had a higher PR = 1.03 (95% CI: 0.78–1.37) for the death outcome than women with less than 8 years of schooling. It is also noteworthy that drinking alcohol weekly had a PR = 1.11 (0.68–1.79) higher for the occurrence of death than mothers who did not drink or drank alcohol only twice a month. Women who did not suffer from violence were effectively associated with neonatal mortality, with PR = 2.9 (95% CI: 0.44–19.13). Likewise, women who had not been diagnosed with high blood pressure [PR = 1.32 (95% CI: 1.10–1.60)], and who had not had multiple pregnancies [PR = 1.34 (95% CI) %: 1.01–1.78)] and had a membrane rupture for more than 24 h [RP = 1.04 (95% CI: 0.79–1.37)] were positively associated with the proven dependent variable.

All the variables related to the care profile had a statistically significant association with neonatal mortality. When the newborn was identified as having an undefined or unclassified sex, there was a statistically significant increase in the likelihood of death (PR = 2.96) when compared to male newborns. Neonates weighing 500 g or less and with a gestational age of less than 24 weeks were approximately five times more likely to die. Not performing mask or balloon ventilation had a higher PR = 1.28 (95% CI: 1.06–1.55) for the outcome studied. Newborns who were not admitted to the Conventional Neonatal Intermediate Care Unit had a PR = 6.63 (95% CI: 5.05–8.70) higher for the outcome studied. Those who were not admitted to the Kangaroo Neonatal Intermediate Care Unit had a statistically significant 73-fold increase in the likelihood of death. It is also noted that the use of antibiotics in neonates with a lifespan of 48 h or less had a higher PR = 2.30 (95% CI: 1.87–2.84) than newborns who did not use the medication (Table 2).

Table 3 shows the final predictive regression model of the determining factors of neonatal mortality in newborns born at term and prematurely, respectively. The analysis shows that the factors most associated with death in term newborns were drug intake by the mother and admission to the Neonatal Intensive Care Unit. For preterm newborns, the predictor variables were the use of cannula ventilation, 1 min Apgar score <7, and admission to the intensive care unit.

## 4. Discussion

### 4.1. Social and Clinical Profile of Mothers of Newborns Being Monitored for Neonatal Care by the Qualineo Strategy

The data from this study point to risk factors for newborn mortality related to maternal social and clinical aspects. When analyzing the PR of the mother’s age variable, it was found that being between 20–29 years old represents a lower probability of neonatal death when compared to the 10–14 age group.

Most of the mothers in this study were aged between 20 and 29, an age group that, from a biological point of view, represents the peak of female fertility and is associated with lower risks of pregnancy complications, given that pregnancy in adolescents (under 15) and advanced maternal age (over 35) can have worse outcomes for mother and baby such as premature birth, preeclampsia, chromosomopathies, and miscarriages [11,12].

A prospective study carried out in Nepal that aimed to analyze neonatal hospital outcomes in an intensive care unit found that premature neonates had three times the risk of neonatal mortality than full-term newborns, and that a low maternal age (<20 years) was a predictor of negative outcomes [13]. Thus, this study proves that the extremes of maternal age can influence the outcomes studied.

Regarding lifestyle habits, this study shows that abstaining from smoking or using illicit drugs is a protective factor in preventing the outcome of death. In addition, weekly alcohol consumption during pregnancy was positively associated with neonatal mortality. It is known that the use of licit and illicit drugs hinders the passage of oxygen to the fetus and can lead to underweight neonates with an increased risk of negative outcomes, such as hospitalization in neonatal units and death [14]. Thomas et al. (2022) evaluated the impact of US state policies controlling the use of these substances during pregnancy on maternal and infant mortality rates and concluded that laws punishing the use of drugs during prenatal care should be supported, since there has been an increase in deaths related to these habits [15].

We found a 32% increase in the likelihood of neonatal death in women who were not diagnosed with hypertension. This comorbidity is one of the maternal clinical conditions that increase the risk of morbidity and mortality for mother, fetus, and newborn. In the scientific literature, the relationship between hypertensive syndromes during pregnancy and negative outcomes for the newborn is widespread. Studies show that, in addition to death, increased blood pressure levels have repercussions in terms of increased admissions to neonatal units; prematurity and the risks associated with it (infection, respiratory difficulties, apnea, hypoglycemia, and convulsions, among others); and the separation of the maternal–neonatal binomial when the pair requires intensive care [15,16,17,18]. A systematic review of 97 studies found an association between chronic hypertension and premature birth, small for gestational age, and stillbirth—conditions that increase neonatal mortality rates and reinforce the relationship between this comorbidity and adverse outcomes [19].

Thus, it is suggested that the results of our study may be related to the better health care that hypertensive pregnant women received, with access to specialized tests that lead to assertive conduct. The results may also be associated with the location of the study, a highly complex maternity hospital, which results in more careful and qualified care. However, it should be noted that the Qualineo Strategy’s neonatal care monitoring form does not identify the presence of other health conditions associated with blood pressure, such as pre-eclampsia, nor does it make it possible to know the severity of the disease, which could result in different data from that presented and a better understanding of the risk factors associated with neonatal death.

Another clinical condition analyzed was the type of pregnancy, where it was found that not having a multiple pregnancy was positively associated with the outcome studied. This contrasts with the risks of twin pregnancies for maternal and neonatal health documented in the literature, such as pre-eclampsia, gestational diabetes mellitus, miscarriage, premature birth, and low birth weight—conditions that can lead to the death of the neonate in the first week of life [19,20]. The review carried out by Whittaker et al. (2023) corroborates the above information by associating greater risks of adverse outcomes with twin pregnancies such as an increased risk of hospitalization in neonatal intensive care units, premature birth, and fetal and neonatal mortality [21].

Similar to that found for the hypertension variable, the lower risk of neonatal mortality observed in twin pregnancies was related to the special care offered to pregnant women with multiple pregnancies treated at the study institution, both during prenatal care and during delivery or cesarean section, in view of understanding the high-risk clinical conditions that can have repercussions on undesirable outcomes. In addition, more attentive obstetric care, with greater preparation of the neonatal care team soon after birth, has repercussions on a safer transition to the extrauterine environment.

The variable “ruptured pouch” refers to the time elapsed in hours from the moment the amnion and chorion membranes have ruptured until birth. One of the main complications of this condition is infection of the amniotic cavity, which is associated with an increase in ruptured pouch time and can compromise the health of both the mother and the fetus/neonate [22,23,24]. In the results presented, a longer ruptured pouch time (greater than 24 h) has a higher PR for the occurrence of newborn death. These data provide support for the creation of actions aimed at reducing the risk of maternal and, consequently, neonatal infection during the care provided. However, the Qualineo Strategy monitoring form does not include the gestational age at which the membranes ruptured, which is important information for defining the conduct of health professionals. In addition, the fetal and neonatal prognosis can change according to the number of weeks of gestation, with the period before 24 weeks being the one with the greatest risk of unfavorable outcomes for the fetus and neonate.

Newborn respiratory distress syndrome is an important variable in worsening the perinatal morbidity and mortality scenario, represents the second leading cause of early neonatal mortality, and is the main cause of NICU admissions in Brazil [3]. Studies have shown a significant association between respiratory distress in newborns and premature birth, cesarean section, birth weight of less than 2500 g, insufficient use of antenatal corticosteroids by pregnant women and an Apgar score of less than 7 [25,26]. A scoping review carried out by Mwita et al. (2021) assessed the impact of the use of corticosteroids by women at risk of preterm birth and found that the majority of studies reported an association between the use of these substances and low neonatal mortality rates [27].

In our study, the non-use of antenatal steroids had a negative association with the outcome studied. Although this result does not initially corroborate results in the literature, pregnant women in the study who did not use antenatal steroids probably did not have a risk of preterm labor; thus the risk of neonatal mortality was reduced compared to women who had their children before term. This is associated with the possibility of an incomplete dose of corticosteroids before preterm birth, which may not have repercussions on the comprehensive protection of newborn respiratory distress syndrome after delivery.

In relation to the type of delivery, neonates born by cesarean section were less likely to die. A similar result was found in a study carried out in Brazil by Serra et al. (2022) and Saloio et al. (2020), where cesarean sections were less likely to cause perinatal death [8,28]. On the other hand, a retrospective study carried out in Jordan presented relevant data associating cesarean sections with adverse conditions for the newborn that increase the chances of death in the first 28 days such as prematurity, low birth weight, fetal distress, low Apgar score, respiratory syndromes, and hospitalization in intensive care units [29].

The protective effect of a cesarean section can be understood by the fact that it is performed in an operating room, a controlled environment in which the pregnant woman remains under the supervision of an obstetrician, anesthesiologist, and nursing staff throughout the perioperative period. Furthermore, when surgery is carried out for real indications of maternal or fetal health, it has good outcomes for the maternal–neonatal binomial and enables a safe birth for the newborn. Another aspect to be highlighted is the health conditions of the mother and fetus in our study, consistent with high-risk pregnancies, which made cesarean section a safe way to deal with the emergencies and comorbidities of the gestational period. Although some studies show a lower relationship between cesarean sections and neonatal mortality, caution is needed when analyzing the “route of delivery” variable, given that vaginal delivery for pregnant women who have no real contraindications for this route is still considered safer in the neonatal transition from the uterus to the extrauterine environment, and provides greater clinical benefits for the mother.

### 4.2. Care and Clinical Characteristics of Newborns Monitored by the Qualineo Neonatal Care Strategy

Neonates with an undefined or unclassified sex were more likely to die in our study. This condition consists of a congenital malformation that makes it impossible to identify the sex of the neonate at birth. The association of congenital anomalies with increased infant mortality rates, especially in the neonatal period, makes early diagnosis essential for planning strategies and allocating resources to specialized health services [30,31].

Birth weight is an important marker of maternal and infant health, revealing aspects related to the quality of prenatal care and encompassing social and economic factors which, alone or together, can lead to a fetus/neonate with inadequate weight for gestational age. Low birth weight is commonly related to prematurity, where both conditions increase the risk of neonatal mortality, as well as infection rates and hospitalization in neonatal units [32,33,34]. A study carried out in Africa with neonates admitted to specialized units reinforces the above; it was identified that neonatal death was associated with extreme low birth weight (40.9%) and very low birth weight (30.5%) [35].

Convergently, this study found a higher probability of death in newborns weighing less than 1500 g, which reinforces the association between this variable and the study’s outcome. Furthermore, it is necessary to understand the other repercussions generated by low birth weight for the newborn and their families, given that those who do not die remain hospitalized for long periods for monitoring and weight gain, are sometimes separated from their parents, have difficulties fighting infections, and have increased chances of developing metabolic syndromes in adulthood, among other consequences [36,37].

With regard to gestational age, the results indicate a higher PR for death in neonates born at less than 31 weeks, with the highest probability of such an outcome being found in extremely premature infants (less than 28 weeks). This finding corroborates results described in other Brazilian studies, where premature birth was shown to be a relevant precedent for perinatal and neonatal mortality [14,17]. A study carried out in sub-Saharan Africa and in Asia identified the most common causes of neonatal deaths in places with high infant mortality in those regions, including infection (40%), prematurity (32%) and respiratory distress syndrome (28%) [38], reinforcing our findings.

In this context, the COVID-19 pandemic has also led to a number of public health problems, including an increase in preterm births. In addition, infection with the SARS-CoV-2 virus has hindered pregnant women’s access to health services, which, coupled with gestational immune vulnerability, has increased morbidity and mortality rates for the mother–child binomial [39,40]. When analyzing the effect of nurse-mediated management of cases of prematurity, one study revealed that actions, such as individual and collective guidance, prenatal nursing consultations, home visits and telephone monitoring, contributed towards preventing prematurity [41]. These data reinforce the role of nurses as fundamental mediators in implementing the new model of obstetric and neonatal care based on good practices that are based on scientific evidence and the humanization of care.

Neonatal resuscitation is a set of specialized actions to help the neonate transition to the extrauterine environment, which has different characteristics to those found inside the womb. As such, resuscitation is an important resource for reducing neonatal mortality, especially early neonatal mortality. To this end, professionals who provide obstetric and neonatal care should be trained on an ongoing basis, an action that is low in cost and results in fewer negative outcomes [42]. In our study, neonates who did not undergo neonatal resuscitation were less likely to die than those who required some assistance in the adaptation process after birth. Similarly, other variables related to this assistance also showed a negative association with the outcome analyzed, including the use of oxygen, the use of cannula ventilation, cardiac massage, and the use of drugs. Thus, not needing neonatal resuscitation, ventilatory support, and cardiac massage is a protective factor for neonatal death. It is therefore understood that neonates who did not need this assistance were born with good vitality and therefore had a lower risk of morbidity and mortality in the first 28 days of life.

Furthermore, it was found that neonates with an Apgar score of less than 7 in the first and fifth minutes were more likely to die. This result is in line with studies in the literature on this score and reinforces the importance of using it for an accurate assessment of the newborn, since the signs assessed represent parameters for determining the quality of resuscitation maneuvers [11,43]. In agreement, a population-based cohort study carried out in Australia showed that an Apgar score of <4 at the fifth minute was prognostic of neonatal mortality and severe and non-severe neurological morbidity [44].

In this study, the majority of neonates had the cord clamped immediately (before one minute), but those who had it clamped later (after one minute) were less likely to die. The literature has shown that, despite the numerous benefits of waiting for the umbilical cord to stop pulsating before clamping it—such as a higher iron intake, higher rates of exclusive breastfeeding and maintenance of body temperature—there are still high rates of early/immediate clamping in maternal and childcare institutions [45,46,47]. In view of this, it can be concluded that although there are current guidelines on late clamping of the umbilical cord, there is still resistance on the part of health professionals to follow these recommendations, who sometimes follow outdated and interventionist practices, leading to greater risks for the health of the mother and the newborn. On the other hand, given the reality of the study site, it can be inferred that the neonates with the most serious clinical conditions required immediate clamping of the umbilical cord in order to receive pediatric care and assess the need for resuscitation maneuvers. Consequently, there is a higher probability of neonatal death in this population, which justifies the variable’s positive association with the outcome.

Regarding hypothermia, the study shows that neonates who did not receive measures to prevent hypothermia in the delivery room had a lower probability of neonatal death. This finding can be explained by the literature, in which the majority of neonates who require measures to maintain body temperature are premature or underweight, and thus present risk factors for interventions at birth and, consequently, morbidity and mortality and hospitalization in neonatal units [48]. Therefore, in view of the need to control or prevent hypothermia, effective and timely actions are necessary, given that a reduction in body temperature can be an aggravating factor for neonates who already have other risk conditions, and thus increase the likelihood of early neonatal death.

In this study, newborns who were not admitted to the conventional care unit or the kangaroo mother care unit were more likely to die, as were those who required admission to the NICU. Newborns admitted to intensive care units have unfavorable clinical characteristics, which can lead to negative outcomes such as death [49]. Singh et al. (2023) showed that early hospitalization and length of stay are significant predictors of mortality in neonatal care units [50].

Neonates who did not use conventional mechanical ventilation were less likely to die. This is probably associated with the neonate’s difficulty in maintaining adequate breathing after birth, a condition that is commonly related to prematurity and maternal and neonatal complications during pregnancy [51,52]. Furthermore, despite the recognized and accepted benefits of conventional mechanical ventilation for establishing a regular breathing pattern, prolonged use is correlated with complications that increase neonatal morbidity and mortality rates such as airway injuries, barotrauma, nosocomial pneumonia, reduced venous return and muscle atrophy [53]. Therefore, qualified and attentive assistance during prenatal care is necessary to identify risk factors for the use of mechanical ventilation after birth, acting mainly to reduce maternal morbidities and prevent prematurity and low birth weight.

The findings of this study indicate that not having a diagnosis of infection resulted in a lower probability of neonatal death. The literature points to neonatal sepsis as a public health problem; although there are advanced technologies that enable early diagnosis and treatment, the infectious condition still represents one of the main causes of neonatal mortality, especially in developing countries such as Brazil [54]. While recognizing and analyzing the main risk factors for sepsis in the neonate are important, it is also necessary to create effective strategies for control and reduction.

In association with neonatal sepsis, there is a need to use antibiotics to treat the condition and improve clinical outcomes. In this sense, we found that neonates who used medication in the first 48 h of life were more likely to die. The potential of early neonatal sepsis to deteriorate the newborn’s health and increase the risk of morbidity and mortality corroborates with a study by Abiy et al. (2023) that assessed neonates diagnosed with sepsis, where the incidence rate of death was 28 per 1000 neonates/day of observation [55]. On the other hand, a study by Oliveira and Sorte (2022) revealed that the majority of newborns treated for neonatal sepsis in a neonatal intensive care unit died by the 7th day of life were associated with the outcomes of a birth weight of less than 1000 g and the presence of hyperthermia and tachypnea [56].

The final predictive regression model identified determining factors for neonatal death. Pregnant women who use drugs, especially illicit drugs, are socially and clinically vulnerable. This reality makes it difficult for them to access health services, and, consequently, prenatal care, which, combined with the maternal and fetal risks inherent in the consumption of illicit substances (placental abruption, prematurity, fetal malformations, low birth weight, sudden death, among others), increases the chance of poor outcomes for the newborn such as death in the first few days of life [38,41].

Admission to the intensive care unit was identified as a determining variable for the outcome of both full-term and premature neonates. This finding corroborates other studies in which intensive care was associated with neonatal death, given that the newborn’s unfavorable clinical conditions (prematurity, congenital malformations, neonatal asphyxia, among others) justify the need for admission to these units and hinder a successful prognosis [57,58,59].

Predictive factors for death in preterm neonates include an Apgar score of less than 7 in the first minute and the use of mechanical ventilation with a cannula, which is seen as an important tool in the care of neonates with respiratory failure; however, prolonged use is described as a risk factor for high morbidity and mortality rates due to complications such as airway injuries and pneumonia [51]. It is therefore necessary for health services and professionals to have in-depth knowledge of the systemic and respiratory repercussions of this invasive therapy and to develop strategies to reduce the risks.

## 5. Conclusions

This study analyzed which of the Qualineo Strategy’s neonatal care indicators are related to the neonatal death outcome. All the neonatal variables analyzed showed a significant association with the death outcome, especially with those that are avoidable such as prematurity, low birth weight and early and late infection. The identification of conditions associated with the outcome of the study make it possible to visualize better strategies for the reality analyzed, which has specific characteristics related to the public served and the care provided. In addition, the data presented reinforce the importance of prenatal care, which is the basis for a healthy pregnancy and an uneventful birth, helping to reduce undesirable outcomes for the newborn such as death and hospitalization in neonatal units. Thus, strategic actions need to be present at various levels of complexity, especially in primary health care.

The study was supported by the generation of important results about the evaluation of the Qualineo Strategy, providing subsidies for the development of effective interventions to improve neonatal health care. There is also a lack of publications analyzing the Qualineo Strategy and its impact on reducing the number of deaths in Brazil. Furthermore, we believe that the study will contribute not only to improving newborn care but also to improving maternal health, given that clinical aspects of the mother interfere with neonatal morbidity and mortality.

However, this study has limitations in relation to data collection using secondary data, with the possibility of omissions due to failures in filling out and typing in the neonatal care monitoring forms. The Qualineo Strategy was implemented at the study collection site in 2017; however, the data only began to be filled in fully and with greater rigor in the spreadsheets obtained for the analysis from 2021 onwards, which made it impossible to understand the risk factors for neonatal mortality in the first years after implementation. Furthermore, the study location is a high-risk maternity hospital that has specificities specific to this type of service, which prevents the results of this study from being projected to other health institutions with different characteristics.

## Figures and Tables

**Table 1:** Prevalence ratio of the death outcome with the social and clinical profiles of the mothers of newborns being monitored for neonatal care by the Qualineo Strategy in 2021 and 2022. Teresina, Piauí, Brazil, 2023.

Variables	Deaths		PR * (95% CI **)	*p*-Value
	No.	%		
Mother’s age (years)				
10 to 14	6	35.3	1.0	-
15 to 19	70	27.0	0.76 (0.39–1.50)	0.438
20 to 29	183	21.4	0.61 (0.32–1.17)	0.137
30 to 39	117	20.4	0.58 (0.30–1.12)	0.104
≥40	31	29.8	0.84 (0.41–1.71)	0.640
Skin color				
White	36	25.0	1.0	-
Black	24	21.4	0.86 (0.54–1.35)	0.506
Brown	280	23.1	0.92 (0.68–1.25)	0.604
Asian/Indigenous	5	20.8	0.83 (0.36–1.91)	0.667
Schooling (years of study)				
<8	43	22.0	1.0	-
≥8	340	22.8	1.03 (0.78–1.37)	0.822
Smoking				
Yes	22	30.6	1.0	-
No	382	22.0	0.72 (0.50–1.03)	0.075
Frequency of alcohol intake during pregnancy				
No drinking or twice a month	382	22.2	1.0	
Weekly	13	24.5	1.11 (0.68–1.79)	0.679
Drug intake				
Yes	8	25.0	1.0	
No	390	22.3	0.89 (0.49–1.64)	0.716
Undergone violence				
Yes	1	7.7	1.0	-
No	386	22.3	2.9 (0.44–19.13)	0.268
Hypertension				
Yes	109	18.4	1.0	-
No	295	24.2	1.32 (1.10–1.60)	0.006
Multiple pregnancy				
Yes	45	17.2	1.0	-
No	355	23.2	1.34 (1.01–1.78)	0.039
Rupture of membranes (hours)				
<18	48	19.9	0.87 (0.66–1.15	0.331
18 to 24	7	13.2	0.58 (0.29–1.16)	0.125
>24	45	23.7	1.04 (0.79–1.37)	0.783
No	304	22.8	1.0	-
Antenatal steroids				
Yes	220	24.3	1.0	-
No	184	20.2	0.83 (0.70–0.99)	0.037
Type of delivery				
Vaginal/Forceps	135	28.2	1.0	-
Cesarean section	273	20.1	0.71 (0.60–0.85)	<0.001

* Prevalence ratio, ** Confidence interval.

**Table 2:** Prevalence ratio of the outcome death with the care and clinical profile of newborns being monitored for neonatal care by the Qualineo Strategy in 2021 and 2022. Teresina, Piauí, Brazil, 2023.

Variables	Deaths		PR * (95% CI **)	*p*-Value
	No.	%		
Gender				
Male	219	22.5	1.0	-
Female	188	21.7	0.96 (0.81–1.14)	0.671
Undefined or Unclassified	6	66.7	2.96 (1.84–4.77)	<0.001
Birth weight (grams)				
≤500	8	80.0	5.69 (3.90–8.30)	<0.001
501 to 999	161	76.3	5.43 (4.32–6.82)	<0.001
1000 to 1499	86	26.8	1.90 (1.44–2.52)	<0.001
1500 to 2499	88	11.0	0.78 (0.58–1.04)	0.095
≥2500	71	14.1	1.0	-
Gestational age (weeks)				
<24	25	86.2	5.05 (3.91–6.52)	<0.001
24 to 27	104	74.8	4.39 (3.47–5.53)	<0.001
28 to 30	83	41.5	2.43(1.86–3.18)	<0.001
31 to 33	62	15.5	0.91 (0.66–1.24)	0.535
34 to 36	68	10.3	0.60 (0.44–0.82)	0.001
≥37	72	17.1	1.0	-
Neonatal resuscitation				
Yes	269	37.0	1.0	-
No	144	12.9	0.35 (0.29–0.42)	<0.001
Use of oxygen				
Yes	251	38.2	1.0	-
No	12	21.4	0.56 (0.34–0.94)	0.027
Use of mask or balloon ventilation				
Yes	133	33.4	1.0	-
No	131	42.8	1.28 (1.06–1.55)	0.011
Use of high-flow nasal cannula				
Yes	222	56.4	1.0	-
No	45	14.1	0.25 (0.19–0.33)	<0.001
Cardiac massage				
Yes	44	71.0	1.0	-
No	224	34.0	0.48 (0.40–0.58)	<0.001
Drug intake				
Yes	40	72.7	1.0	-
No	227	34.2	0.47 (0.39–0.57)	<0.001
1-min Apgar score				
<7	276	42.2	1.0	-
≥7	125	11.1	0.26 (0.22–0.32)	<0.001
5-min Apgar score				
<7	100	58.8	1.0	
≥7	301	18.6	0.32 (0.27–0.37)	<0.001
Cpap in the delivery room				
Yes	89	14.0	1.0	-
No	270	30.1	0.71 (0.60–0.85)	<0.001
Umbilical cord clamping time				
<1 min	362	25.8	1.0	-
≥1 min	34	9.6	0.37 (0.26–0.52)	<0.001
Measures to avoid hypothermia in the delivery room				
Yes	261	29.1	1.0	-
No	145	16.4	0.56 (0.47–0.57)	<0.001
Admission to the Neonatal Intensive Care Unit				
Yes	345	38.8	1.0	-
No	61	7.2	0.18 (0.14–0.24)	<0.001
Admission to the Conventional Intermediate Care Unit				
Yes	54	5.9	1.0	-
No	332	39.4	6.63 (5.05–8.70)	<0.001
Admission to the Kangaroo Intermediate Care Unit				
Yes	4	0.6	1.0	-
No	369	40.9	73.74 (27.65–196.61)	<0.001
Conventional mechanical ventilation				
Yes	209	37.5	1.0	-
No	165	14.2	0.38 (0.32–0.45)	0.001
Use of surfactant				
Yes	177	47.8	1.0	-
No	229	16.0	0.33 (0.28–0.39)	<0.001
Early infection				
Yes	223	30.6	1.0	-
No	179	16.4	0.54 (0.45–0.64)	<0.001
Late infection				
Yes	187	34.7	1.0	-
No	205	16.9	0.49 (0.41–0.58)	<0.001
Use of antibiotics in the first week of life				
Not used	127	15.5	1.0	-
≤48 h	135	35.7	2.30 (1.87–2.84)	<0.001
>48 h and ≤72 h	32	24.4	1.58 (1.12–2.22)	0.009
>72 h and ≤7 days	65	20.7	1.34 (1.02–1.75)	0.035
>7 days	54	26.2	1.69 (1.28–2.24)	<0.001

* Prevalence ratio, ** Confidence interval.

**Table 3:** Predictive regression model of the determinants of neonatal mortality in term newborns. Teresina, Piauí, Brazil, 2023.

Variables	PR * (95% CI **)	Standard Error	*p*-Value
Newborn on term			
Drug intake	2.89 (1.74–4.81)	0.75	<0.001
Admission to the Neonatal Intensive Care Unit	3.52 (1.40–8.85)	1.66	0.007
Preterm newborn			
Use of cannula ventilation	2.89 (2.22–4.64)	0.60	<0.001
1 min Apgar <7	1.51 (1.09–2.08)	0.25	0.016
Admission to the Neonatal Intensive Care Unit	1.40 (1.09–1.79)	0.18	0.012

* Prevalence ratio, ** Confidence interval.

## Data Availability

The data used are available within the manuscript.
